# Motivational Variables as Moderating Effects of a Web-Based Mental Health Program for University Students: Secondary Analysis of a Randomized Controlled Trial

**DOI:** 10.2196/56118

**Published:** 2024-07-03

**Authors:** Maria Hanano, Leslie Rith-Najarian, Elizabeth Gong-Guy, Denise Chavira

**Affiliations:** 1 University of California, Los Angeles Los Angeles, CA United States; 2 Strive Weekly Los Angeles, CA United States

**Keywords:** web-based intervention, internal motivation, depression, anxiety, self-determination theory, mental health, university students, university, students, web-based, analysis, randomized controlled trial, self-guided

## Abstract

**Background:**

Self-guided web-based interventions have the potential of addressing help-seeking barriers and symptoms common among university students, such as depression and anxiety. Unfortunately, self-guided interventions are also associated with less adherence, implicating motivation as a potential moderator for adherence and improvement for such interventions. Previous studies examining motivation as a moderator or predictor of improvement on web-based interventions have defined and measured motivation variably, producing conflicting results.

**Objective:**

This secondary analysis of data from a randomized controlled trial aimed to examine constructs of motivation as moderators of improvement for a self-guided 8-week web-based intervention in university students (N=1607).

**Methods:**

Tested moderators included internal motivation, external motivation, and confidence in treatment derived from the Treatment Motivation Questionnaire. The primary outcome was an improvement in depression and anxiety measured by the Depression Anxiety Stress Scale-21.

**Results:**

Piecewise linear mixed effects models showed that internal motivation significantly moderated symptom change for the intervention group (*t*_1504_*=*–2.94; *P=*.003) at average and high (+1 SD) motivation levels (*t*_1507_=–2.28; *P=*.02 and *t*_1507_=–4.05; *P<*.001, respectively). Significant results remained even after controlling for baseline severity. The results showed that confidence in treatment did not significantly moderate symptom change for the intervention group (*t*_1504_*=*1.44; *P*=.15). In this sample, only internal motivation was positively correlated with service initiation, intervention adherence, and intervention satisfaction.

**Conclusions:**

The combination of a web-based intervention and high or moderate internal motivation resulted in greater improvement in the total Depression Anxiety Stress Scale-21 score. These findings highlight the importance of conceptually differentiating motivation-related constructs when examining moderators of improvement. The results suggest that the combination of a web-based intervention and high or moderate internal motivation results in greater improvement. These findings highlight the importance of conceptually differentiating motivation-related constructs when examining moderators of improvement. To better understand the moderating role of internal motivation, future research is encouraged to replicate these findings in diverse samples as well as to examine related constructs such as baseline severity and adherence. Understanding these characteristics informs treatment strategies to maximize adherence and improvement when developing web-based interventions as well as allows services to be targeted to individuals likely to benefit from such interventions.

**Trial Registration:**

ClinicalTrials.gov NCT04361045; https://clinicaltrials.gov/study/NCT04361045

## Introduction

### Background

Self-guided web-based mental health interventions have increased in popularity as an alternative service option for young people, and many have proven effective in reducing an array of mental health problems [1]. Delivering such interventions to university students is particularly advantageous, as they address many barriers associated with traditional in-person campus services—concerns about confidentiality, scheduling issues, preference for self-reliance, etc [2,3]—and can be delivered on a large-scale prevention level. A particularly unique feature of web-based interventions is their ability to offer users increased autonomy to interact with the intervention according to their own preferences and needs, with less oversight [4]. However, the direct drawback of this is that self-guided web-based mental health programs have low adherence and completion rates [5-7]. According to Eysenbach’s law of attrition [8], users of web-based interventions receive variable doses of the intervention due to less oversight and more autonomy. Consequently, any observed effects in symptom improvement favoring web-based interventions may be driven by those who actually remain engaged with the program. We are thus left with uncertainty: Does the web-based intervention itself lead to improved symptoms or are there characteristics of a subset of individuals that are driving effects? With web-based mental health interventions being substantially newer than in-person psychotherapies, there has increasingly been a call to identify intervention response moderators in order to better understand for whom these interventions work [9-11].

Given the self-guided nature of web-based interventions, motivation is likely an important moderator to consider. One of the prominent theories on motivation is self-determination theory (SDT), a theory concerned with human motivation, which assumes that individuals have an innate need to develop and grow psychologically [12,13]. As such, they theorize that an individual’s drive to attain different types of goals is determined by 3 key needs that advance our growth: competence, autonomy, and relatedness. According to SDT [12,13], there is a spectrum of motivation ranging from external to intrinsic: external (ie, drive to reduce negative consequences or punishments or secure tangible rewards), introjective (ie, to reduce internalized pressure, shame, and guilt), identifying (ie, drive toward goals and values that are identified as personally important), integrated (ie, drive to act in congruence with values that are core to one’s sense of self), and intrinsic (ie, drive by interest, curiosity, satisfaction, and enjoyment). For the purposes of our paper, SDT provides a useful framework to conceptualize motivation, and we define internal motivation as all nonexternal types of motivation (ie, introjective, identifying, integrated, and intrinsic).

SDT research has found that the various kinds of motivation differentially predict various health behaviors (ie, physical exercise and dieting) [14] as well as greater treatment adherence and progress toward mental health interventions [13,15]. According to student feedback from an open trial of a web-based intervention [16], motivational difficulty was one of the most common self-reported barriers to using the intervention. One study examining adults with alcohol use disorder enrolled in an alcohol treatment program found that those with more autonomous (ie, identified or integrated motivation) reasons for engaging with the program had more regular attendance and involvement [17]. Another study that tested positive psychology self-help interventions found that university students with more intrinsic motivation experienced greater improvement after intervention [18]. The authors concluded that in order for self-help interventions to be maximally effective, users need to (1) be receiving empirically supported intervention practices and (2) have their own motivation to use the intervention. Taken together, these SDT studies suggest that internal motivation is an important moderator of treatment engagement and response for in-person interventions.

Studies that have examined motivation as a moderator or predictor for web-based interventions have measured motivation differentially and produced conflicting results. For example, a study testing a web-based intervention for marijuana use in university students tested if effects were moderated by a readiness-to-change measure, which the authors selected to “assess level of motivation to change” [19]. The results of the study suggested moderation by the action subscale (ie, taking actual steps to change) such that higher action scores predicted greater symptom improvement but no significant moderation by the problem recognition subscale [19]. A different study of a web-based cognitive behavioral therapy intervention for adults operationalized “motivation” using a measure [20] with 4 domains—readiness to change, distress from symptoms, social support, and doubt concerning participation—and found that “high motivation” predicted less depression improvement [21]. However, in a study of a web-based relaxation intervention for adults with mild to moderate anxiety levels, there was no relationship between baseline “internal motivation” and postintervention reduction of stress symptoms [22]. In that same study, high baseline “external motivation” (eg, feeling pressured by others to get help) predicted worsened stress symptoms by postintervention [22]. In summary, previous web-based intervention research has used variable definitions and measures of motivation, which could be driving the divergent results.

### This Research

Given the mixed findings of research examining motivation as a moderator of web-based mental health intervention effects, there is a need for web-based intervention research that operationalizes the construct of motivation using a more standardized definition. In contrast, SDT research has used a standardized definition of motivation and has more consistently found motivation to be a moderator of intervention response and engagement, but only for in-person interventions. It has already been recommended to consider SDT when developing and testing digital mental health apps, given that intrinsic motivation is hypothesized to drive adherence behaviors [23].

In service of this aim, we selected the Treatment Motivation Questionnaire (TMQ) [17] to operationalize our moderators. The TMQ was developed specifically to measure SDT’s motivation constructs in the context of treatment-seeking [17]. The TMQ has an internal motivation scale, an external scale, and a confidence in treatment scale. This third scale is included because the measure developers argue that in order to be motivated to seek treatment, individuals must have some confidence in the treatments’ competence and ability to provide successful outcomes [17]. Indeed, the construct of confidence in treatment was found to positively correlate to internal motivation, involvement in treatment, less dropout, and number of activities completed and was negatively correlated to external motivation [17]. Prior web-based intervention research has perhaps conflated these separate, yet related motivational constructs, namely, internal motivation for help-seeking, external motivation for help-seeking, and expectation of the intervention to be helpful. Using the 3 subscales of the TMQ as moderators would allow us to simultaneously test these constructs in one study. As far as we are aware, the TMQ and its 3 subscales have never been used within web-based mental health intervention studies.

To test motivation as a moderator of symptom improvement for web-based intervention users, we used data from a recently completed randomized controlled trial of a self-guided intervention, StriveWeekly [24]. StriveWeekly targets anxiety and depression in university students, given that these are the 2 most common mental health concerns for this population [25,26]. The main results of the randomized controlled trial showed that the intervention effectively reduced symptoms of anxiety and depression [24]. We planned a priori to test 3 baseline variables as moderators: internal motivation for treatment, external motivation for treatment, and treatment expectations. The primary aim of this study is to present these moderation analyses. Such analyses will allow us to determine if symptom improvement is driven by the intervention, baseline motivation, or a combination of both. The secondary aim was to conduct exploratory analyses of how SDT motivation constructs relate to participant characteristics and intervention-related variables.

## Methods

### Participants and Procedures

Participants were at least 18 years of age and were undergraduate, graduate, and professional students at a large public university in Southern California. Recruitment strategies included emails (via the registrar to all enrolled students and over departmental email lists), flyers around campus, social media, and announcements in psychology courses granting research study participation credits. Participation was voluntary, and those participating were given the choice of compensation, either entry into a US $10-$100 gift card drawing or course credit. The only a priori exclusion criterion was concurrent enrollment in a web-based anxiety and depression treatment study on campus, resulting in 9 participants being excluded. We also excluded participants with invalid data reporting (eg, inconsistency in cross-validation item pairs and straight-lined responses), resulting in 18 students being excluded. During the course of the trial, 10 participants withdrew. The final sample included in data analyses was 1607.

During a 3-week period, recruitment materials directed participants to an enrollment website link. Informed consent was obtained web-based and then participants began the baseline survey. Within 72 hours thereafter, participants were informed via email of their condition, assigned using an electronic random number generator. Participants in the intervention condition received a verification code to access the web-based platform. The 8-week intervention phase was followed by a postintervention survey, which remained open for 2 weeks for participants in both conditions. Two weeks after completion of the postintervention survey, students in the waitlist condition were granted access to the web-based platform. We also administered a 3-month follow-up survey to all participants who completed the postintervention, though data from this last survey were included in our statistical models solely for missing data estimation.

For the duration of the study, participants in both conditions were allowed to access any other services on- or off-campus. We also delivered a safety follow-up and service referral protocol (enacted by 5 graduate student–level clinicians) to students endorsing suicidal ideation (SI) in any survey (per Patient Health Questionnaire-9’s ninth item [27]). We did not exclude students receiving concurrent services or the additional support, rather we controlled for such differences in our statistical analyses.

### Conditions

#### Intervention Group

Participants first set up their accounts, which included selecting the program brand pathway, setting reminder emails, scheduling preferences, and a goal-setting activity. During the 8-week intervention, students received an email every Monday, which directed them to the section of the web-based platform with the current module’s content. Each module presented a skill (eg, mindfulness and physical exercise) and provided psychoeducation, practice instructions, a list of practice activities, tips and suggestions, and a planning section for participants to tentatively commit to practice activities on a checklist and brainstorm ideas to overcome anticipated barriers to practice. Throughout the week, participants could log any skills they practiced and rate their corresponding mood or stress levels. Participants could earn digital medals for logging more activities, weekly prize raffle entry for submitting any end-of-the-week check-in, and grand prize entry for completing practice for all 8 modules. There was also a “Campus” section on the platform, which provided relevant campus resources, a notification center with campus-specific updates, and an anonymous livestream of all campus users’ activity. For additional details about the intervention content, please see Rith-Najarian et al [24].

#### Waitlist Group

Participants in this group were not contacted during the 8-week intervention until we emailed them with the postintervention survey link. Access to the web-based intervention was provided only if they completed the postintervention survey.

### Measures

#### Demographic Information

Per our consent form, student records were used to collect demographic information such as sex (male or female), student status (undergraduate, graduate, or professional), age, and ethnicity or race (per the Common Data Set categories [28]).

#### Self-Reported Service Use

Students were asked to indicate past and current use of health-related services and resources on- or off-campus using a checklist of common resources or services (eg, counseling center). These questions were included in the baseline and postintervention surveys.

#### Primary Outcome

Our primary symptom outcome measure was the 21-item version of the Depression Anxiety Stress Scale (DASS-21), which assesses self-reported symptoms related to depression, anxiety, and stress. The DASS-21 has demonstrated high internal consistency (0.83-0.90) and good construct validity in university student samples [29]. Internal consistency of the DASS-21 using our sample at baseline was adequate to good (total score: α=.92, depression subscale: α=.89, and anxiety subscale: α=.79). We had previously found [24] that the DASS-21 did not have a good internal factor structure, and thus, we did not examine it on its own. DASS-21 total symptom scores were used.

#### Moderators

The TMQ [17] is a measure that assesses participants’ reasons for initiating treatment and their expectations for completing the program. The TMQ has two motivation scales: (1) internal motivation (eg, “I really want to make some changes in my life.”) and (2) external motivation (eg, “I came to treatment now because I was under pressure to come.”). The TMQ also has a “confidence in treatment” scale (eg, “I am confident this program will work for me.”) and a “help-seeking” subscale (eg, “I accept the fact that I need some help and support from others to beat my problem”). The TMQ has predicted intervention completion in other research studies, for example, in-person alcohol treatment [17] and web-based stress treatment [22]. For this study, questions were minimally adapted to apply to a web-based mental health promotion program instead of an in-person treatment (see Multimedia Appendix 1 for adapted measure). Questions from the help-seeking subscale were removed, as they relate directly to interactions with other treatment participants, which was not applicable to our study design. The TMQ was included in the baseline survey only. Internal consistency was adequate for the TMQ total (α=.80), TMQ internal motivation (α=.87), and TMQ confidence in treatment (α=.74) but not for TMQ external motivation (α=.56).

#### Postintervention Variables

To measure account setup status and adherence, behavioral data from participants’ accounts were collected from their web-based program accounts. Account setup was defined in a binary way, with “yes” being coded if a participant completed the account setup process and verified their email in order to receive intervention email communications. Adherence was defined by the number of weeks (0-8) for which the participant had logged skills practice at least once for the given module. We measured satisfaction by adapting the Program Satisfaction Questionnaire [30], which has previously shown high internal consistency and concurrent validity in intervention research [31,32]. Internal consistency of the 5-item satisfaction scale in our sample was excellent (α=.91). We assessed self-reported barriers encountered during intervention use using an original 10-item measure called the Digital Intervention Barriers Scale (DIBS; [33]). On the DIBS, participants were asked how much they agree on a scale of 1-5 that they encountered a given barrier (eg, “I had technical problems”). In our sample, the DIBS showed good internal consistency (α=.77). Finally, participants completed a checklist of self-identified motivators during the program (eg, weekly prize drawings). See Multimedia Appendices 2 and 3 for these questionnaires.

### Statistical Software and Analyses

#### Preliminary Analyses and Covariate Selection

All preliminary analyses were run in SPSS (version 25; IBM Corp). Differences by condition or dropout status were assessed through independent 1-tailed *t* tests (for continuous variables, eg, DASS-21 scores) or chi-square analyses (for dichotomous variables, eg, ethnicity). For 1-tailed *t* tests, we reported the test statistic for which equal variance was assumed unless the Levene test was significant. We previously identified [24] appropriate covariates for our linear mixed effects (LME) models as (1) gender and (2) baseline SI based on between-group differences in baseline DASS-21 scores.

#### Tests of Moderation

Piecewise LME models were run in R (R Foundation for Statistical Computing) using the multilevel package [34] and accounted for missing data with restricted maximum likelihood estimation, allowing all participants to be included in the analyses. Participant intercepts and time slopes were treated as random effects. Each of the 3 moderators (ie, internal motivation, external motivation, and confidence in treatment) was tested in its own model. The main model included fixed effect terms for group (with intervention condition as the reference group), time, group-by-time interaction, covariates, and covariate-by-time interactions in predicting DASS-21 total scores. To this model, we added the moderator by group-by-time interaction and all lower-order interactions. To transform the moderator into a categorical variable, scores that were 1 SD below the mean or less were coded as “low,” scores 1 SD above the mean or more were coded as “high,” and all scores in between were coded as “average.” In the presence of a significant moderator by group-by-time term, we calculated simple slopes using the *reghelper* package [35] and examined tests of time from baseline to postintervention. If simple slope analyses were also significant, we ran additional LME models for that respective moderator as predicting DASS-21 depression and DASS-21 anxiety as separate outcomes.

#### Effect Sizes

For ease of calculating and interpreting effect sizes for any significant moderator, models were run again, treating the moderator as a categorical rather than a continuous variable. Then, estimated marginal means were calculated with the *emmeans* package for R [36]. Within-group effect sizes were calculated using this formula:



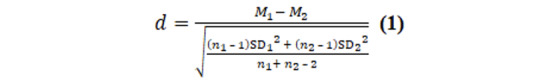



Between-group effect sizes were calculated using Morris’ [37] recommended formula for reducing bias while maximizing precision in pretest-posttest control group designs (*i*=intervention and *w*=waitlist).



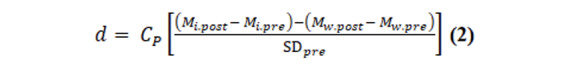





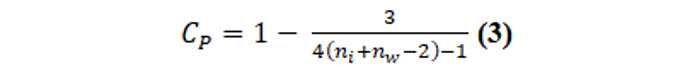





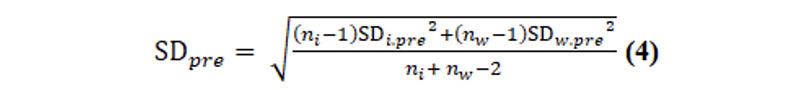



### Ethical Considerations

The main trial from which the presented data were collected was approved by the University of California, Los Angeles’ institutional review board (17-000761). The informed consent was conducted on the web and allows for secondary analysis to be conducted without additional consent from participants. Students who endorsed SI underwent safety follow-up conducted by trained graduate student clinicians as well as were connected with mental health services as needed. Data were collected and stored on an encrypted and secured campus platform and were limited to study staff listed on the institutional review board. When data were initially collected, they were associated with participants’ first names and emails. However, once data sets were created, the data were deidentified, and participants were identified using participants’ IDs. Participant identifying information was then deleted from the secured campus platform, and data sets were stored on password-protected spreadsheets. Participants had the option of selecting 1 of 2 compensation choices for completing the baseline survey, either gift card drawings or research study course credits. Gift card drawings included 1 US $100 and 10 US $10 gift cards per survey. Participants were compensated using gift cards for completing posttest and follow-up surveys.

## Results

### Participant Sample

**Figure 1:**
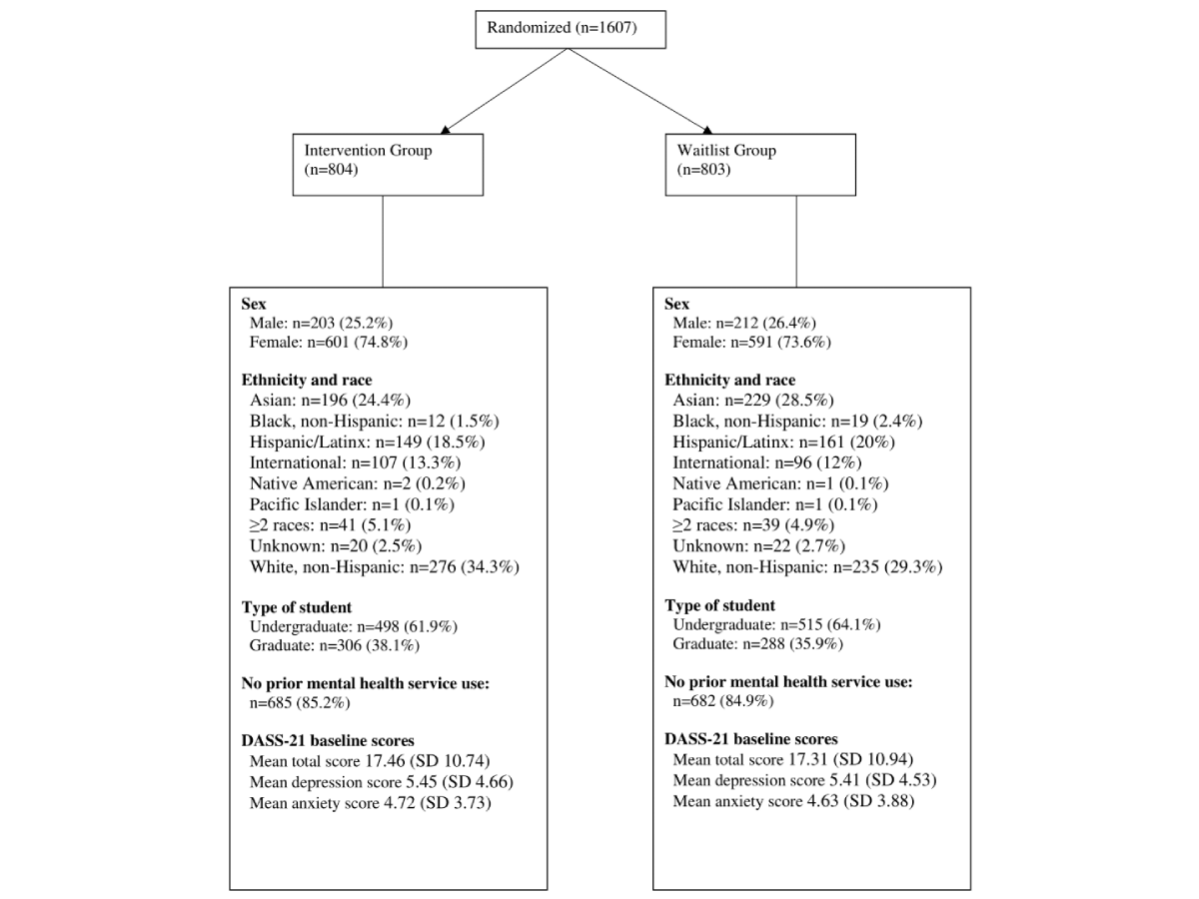
Demographic composition and baseline characteristics by condition in a randomized controlled trial of a self-guided web-based intervention for depression and anxiety for university students (data originally reported by Rith-Najarian [24]). DASS-21: Depression Anxiety Stress Scale-21.

The sample consisted of 1607 ethnically and racially diverse participants, of which 74.2% (n=1192) were female, and 63% (n=1012) were undergraduate students, with a mean age of 22.8 (SD 5.56) years. See Figure 1 for demographic details of students by condition.

### Preliminary Analyses

At baseline, there were no differences between conditions in DASS-21 total or subscale scores (*P*=.62-.85), demographic variables (*P=*.34-.64), baseline SI status (*P*=.31), resource use at baseline (*P*=.94), resource use by postintervention (*P*=.94), or moderator variables (internal motivation: *P=*.56, external motivation: *P*=.42, and confidence in treatment: *P*=.97). Correlations between DASS-21 total and moderators at baseline are presented in Table 1.

Next, we assessed for differences by dropout status. Comparing participants who completed the postintervention survey versus those who had not, we found no differences in DASS-21 scores (*P=*.23-.47), moderator variables (*P=*.68-.76), or the baseline SI covariate (*P*=.39). There was however a statistically significant difference in research dropout by sex (*χ*^2^_1_=14.7; *P<*.001), with 35.5% (n=423) of female students dropping out versus 46.3% (n=192) of male students. There was also a difference in research dropout by condition; 47.1% (n=378) of students in the intervention group dropped out versus 38.5% (n=309) in the waitlist group, such that waitlist participants were disproportionately retained in the postintervention survey (*χ*^2^_1_=12.04; *P*<.001). Examining each condition separately, there were no differences in baseline DASS-21 scores between postintervention completers versus dropouts in the intervention group (*P=*.46-.72) or the waitlist group (*P=*.18-.60).

Finally, we checked for multicollinearity among variables by running separate linear regressions that included both covariates (baseline SI and gender), condition, and each moderator as predictors of change in DASS-21 scores. Variance inflation factor scores for all regressions were between 1.00 and 1.06, suggesting an absence of multicollinearity per the common variance inflation factor <10 cutoff [38].

**Table 1:** Baseline correlations of TMQ^a^ subscales and total scores on the DASS-21^b^ in a randomized controlled trial of a self-guided web-based intervention for depression and anxiety for university students (N=1607).

Variables	DASS-21 total baseline	TMQ external total	TMQ internal total	TMQ confidence total
DASS-21 total baseline	N/A^c^			
TMQ external total	.21^d^	N/A		
TMQ internal total	.46^d^	.23^d^	N/A	N/A
TMQ confidence total	–.20^d^	–.06^e^	–.02	N/A

^a^TMQ: Treatment Motivation Questionnaire.

^b^DASS-21: Depression Anxiety Stress Scale-21.

^c^N/A: not applicable.

^d^*P*<.001.

^e^*P*=02.

### Primary Analyses

#### Testing Moderators of Intervention Outcome Effects

There was a significant group-by-group by time interaction when examining TMQ internal motivation as a moderator of change on total DASS-21 scores (*t*_1504_*=*–2.94; *P=*.003). Simple slope tests of time were significant for the intervention group at average motivation levels (*t*_1504_=–3.41; *P<*.001) and at high (+1 SD) motivation levels (*t*_1504_=–5.39; *P<*.001) but not at low motivation levels for the intervention group nor at any motivation levels for the waitlist group. The remaining 2 moderator models failed to produce significant 3-way interactions: TMQ external motivation (*t*_1504_*=*0.66; *P*=.51) and TMQ confidence in treatment (*t*_1504_*=*1.44; *P*=.15). To control for the false discovery rate across the 4 models, we calculated the Benjamini-Hochberg critical value [39] as *P*=.012 for the 3-way interaction term with the smallest *P* value, and the TMQ internal motivation model adequately met this criterion.

Given that intervention effects on total symptoms were moderated by internal motivation, we ran models predicting change in depression and anxiety symptoms separately. Predicting change in depression subscale scores, there was a significant group-by-time by motivation interaction (*t*_1504_*=*–2.67; *P=*.008), with simple slopes of time being significant only for the intervention group at average motivation levels (*t*_1504_=–2.28; *P=*.02) and at high (+1 SD) motivation levels (*t*_1504_=–4.05; *P<*.001). Predicting change in anxiety subscale scores, there was a significant group-by-time by motivation interaction (*t*_1504_*=*–2.69; *P=*.007), with simple slopes of time being significant only for the intervention group at average motivation levels (*t*_1504_=–3.66; *P<*.001) and at high (+1 SD) motivation levels (*t*_1504_=–5.33; *P<*.001). Treating internal motivation as a categorical variable (low, average, and high), the 3-way interaction terms remained significant (*P*s=.005-02). Table 2 presents the within-group and between-group effect sizes for change in DASS-21 scores by condition and internal motivation levels.

**Table 2:** Effect sizes^a^ and estimated marginal means by condition and internal motivation level in a randomized controlled trial of a self-guided web-based intervention for university students.

Outcome and motivation		Intervention				Waitlist					Between-group *d*
		n (%)	Pre, mean (SE)	Post, mean (SE)	*d*	n (%)	Pre, mean (SE)	Post, mean (SE)	*d*		
**Depression**											
	High^b^	148 (18.4)	8.30 (0.31)	5.93 (0.42)	0.63	131 (16.3)	8.26 (0.33)	7.77 (0.43)	0.13	0.50	
	Average^c^	524 (65.2)	5.24 (0.16)	4.54 (0.22)	0.19	526 (65.5)	5.12 (0.16)	5.01 (0.21)	0.03	0.16	
	Low^d^	128 (15.9)	3.42 (0.33)	3.36 (0.46)	0.02	143 (17.8)	3.57 (0.31)	3.35 (0.41)	0.06	–0.04	
**Anxiety**											
	High	148 (18.4)	6.96 (0.28)	4.81 (0.36)	0.64	131 (16.3)	6.98 (0.3)	6.51 (0.37)	0.14	0.49	
	Average	524 (65.2)	4.46 (0.15)	3.69 (0.19)	0.23	526 (65.5)	4.51 (0.15)	4.27 (0.18)	0.07	0.15	
	Low	128 (15.9)	3.50 (0.30)	3.40 (0.39)	0.03	143 (17.8)	2.87 (0.29)	2.59 (0.35)	0.09	–0.05	
**Total**											
	High	148 (18.4)	24.61 (0.75)	18.30 (1.03)	0.70	131 (16.3)	25.01 (0.80)	23.85 (1.04)	0.13	0.56	
	Average	524 (65.2)	16.88 (0.40)	14.96 (0.54)	0.21	526 (65.5)	16.85 (0.40)	16.69 (0.51)	0.02	0.18	
	Low	128 (15.9)	12.55 (0.80)	12.31 (1.11)	0.03	143 (17.8)	11.66 (0.76)	11.08 (0.99)	0.06	–0.05	

^a^Effect sizes are based on marginal means.

^b^High: +1 SD Treatment Motivation Questionnaire–internal scores.

^c^Average: mean Treatment Motivation Questionnaire–internal scores.

^d^Low: –1 SD Treatment Motivation Questionnaire–internal scores.

#### Post Hoc Probing of Baseline Symptom Severity as a Confounding Variable

Visual examination of estimated marginal means in Table 2 suggested that internal motivation could be affected by baseline severity. Thus, we ran a post hoc analysis to rule out this possibility.

First, we ran another set of LME models that treated baseline DASS-21 scores as a predictor of postintervention DASS-21 scores rather than using a repeated measure dependent variable of DASS-21 scores. Thus, models did not include an effect of time, rather we examined the group by motivation interaction. Of note, because baseline DASS-21 scores were not included within the dependent variable, fewer missing cases could be imputed for postintervention DASS-21 scores; and thus, the sample size was smaller (n*=*992). Over and above the baseline symptom terms, the models produced significant group by motivation interaction terms in predicting postintervention: total symptoms (*t*_985_*=*–3.32; *P*<.001), depression symptoms (*t*_985_*=*2.50; *P*=.01), and anxiety symptoms (*t*_985_*=*3.34; *P*<.001). Thus, regardless of baseline symptom severity, internal motivation still moderated intervention effects.

Second, we divided the internal motivation scale into two subscales: (1) identifying internal reasons for motivation (eg, “I chose this program because I think it is an opportunity for change”; items 1, 5, 9, 11, 15, 17, and 19) versus the remaining items about (2) introjective internal reasons for motivation (eg, “I feel so guilty about my problems that I have to do something about it”). We conceptualized the introjective motivation items as more directly confounded with symptom severity, given that the negative affect aspects (eg, guilt and worry) of the items overlap with diagnostic criteria for mood and anxiety disorders. We found that there was a significant group-by-time interaction for both the LME models testing: the introjective items as a moderator (*t*_1507_*=*–2.51; *P=*.01) as well as the identifying items (ie, those hypothetically less confounded with symptom severity) as a moderator (*t*_1507_*=*–2.66; *P=*.008). Again, simple slopes of time for both LME models were significant only for the intervention condition at average and high motivation levels (*t*_1507_=–5.39 to –2.66; *P=*.008 to *P<*.001). Thus, intervention effects were still moderated by internal motivation as measured by items unrelated to symptom severity.

### Post Hoc Exploratory Analyses

#### Internal Motivation as a Predictor of Service Initiation

We assessed if internal motivation predicted students accessing the web-based intervention itself or other campus services. Using binary logistic regression predicting account setup within the intervention condition (n*=*800), we found that students with higher motivation were more likely to complete setting up their account (Wald *χ*^2^_798_=10.46; β=.02; *P*=.001). In contrast, the other 2 moderator variables did not predict account setup in their own regression models (*P*=.18-72). Next, we ran a binary logistic regression predicting other campus mental health service initiation (ie, added use from baseline to postintervention) in either condition (n*=*911) by internal motivation (high vs not high), condition, and the internal motivation by condition interaction. We found that there was a significant interaction (Wald *χ*^2^_907_=5.66; β=–1.13; *P*=.02), such that for those in the waitlist group, the odds of initiating services were 3.98 greater for those with high motivation, whereas for the intervention group, the odds of initiating services was 1.29 higher for those with high motivation. Again, binary regression models with the other 3 moderator variables produced no significant main effects or interactions in predicting campus service initiation (*P*=.19-.80).

#### Correlates of Motivational Variables at Postintervention

Within the intervention condition (n=804), we assessed if internal motivation related to postintervention variables (ie, adherence, satisfaction, and barriers) differently than did the other tested moderator variables. See Table 3 for all correlations. Although the magnitude was small, internal motivation was significantly and positively correlated with intervention adherence and intervention satisfaction. External motivation was significantly and negatively correlated with intervention adherence. Confidence in treatment had a significant positive correlation with treatment satisfaction and a significant negative correlation with the DIBS self-reported barriers encountered.

**Table 3:** Correlations between motivation construct variables with adherence, satisfaction, and barriers variables in a randomized controlled trial of a self-guided web-based intervention for depression and anxiety for university students (n=804).

Variables	Adherence	Satisfaction	Barriers
TMQ^a^ internal	.09^b^	.13^c^	.05
TMQ external	–.10^d^	–.03	.08
TMQ confidence	.05	.32^e^	–.29^e^

^a^TMQ: Treatment Motivation Questionnaire.

^b^*P*=.02.

^c^*P*=.01.

^d^*P*=.006.

^e^*P*<.001.

## Discussion

### Summary of Findings

Considering the lack of consensus on the moderating effect of motivation for web-based mental health interventions, there is a need to examine motivation using standardized definitions that have been validated in the literature. The primary aim of this study was to test SDT’s 3 motivation constructs—internal motivation, external motivation, and confidence in treatment—as moderators of the effects of a web-based mental health intervention on anxiety, depression, and total symptom change. Our secondary aim was to explore how the 3 motivation constructs relate to participant characteristics and postintervention variables such as adherence and campus service initiation. Such investigation could help us better understand the relative benefit of such self-guided interventions on university campuses, depending on which individual differences predict more improvement in internalizing symptoms.

### Implications

In this study, we found that internal motivation moderated intervention effects, whereas external motivation and confidence in treatment did not have an impact on clinical outcomes. Students with high internal motivation experienced moderately sized effects in the intervention condition relative to the waitlist condition for depression (*d=*0.50), anxiety (*d=*0.49), and total symptoms (*d*=0.56). For students with average motivation, they also did significantly better in the intervention condition relative to the waitlist, but effect sizes were minimal (*d*s from 0.15 to 0.18). For students with low motivation in the intervention condition, there were no differences in symptom change between conditions. The results suggest that moderate to high internal motivation combined with enrollment in a mental health intervention leads to more symptom change. Our results also indicate the inverse that in the absence of an intervention (ie, being assigned to a waitlist group), high internal motivation did not predict significantly more symptom change. Only for students in a self-guided, skill-based web-based intervention, did internal motivation predict greater symptom improvement. A strength of our investigation is a set of post hoc analyses that were conducted to rule out the possibility that the moderating effect of internal motivation was due to overlap with baseline severity. Based on these findings, it would appear that regardless of the severity of symptoms, high internal motivation coupled with an intervention can lead to symptom improvement. An additional strength of this study was that we tested a measure of motivation that is standardized and based on theory. Confidence in treatment was not significantly predictive of symptom improvement. Additionally, external motivation also did not significantly predict symptom improvement. It is important to note that the external motivation TMQ subscale did not show adequate internal consistency, and as such, we cannot confidently make conclusions about external motivations’ role as a moderator of improvement. The results regarding external motivation should be interpreted with caution and must be replicated in other studies using a more reliable measure of external motivation. Ultimately, this study provides support for internal motivation as a moderator of self-guided web-based intervention effects on anxiety, depression, and total symptom outcomes.

Exploratory analyses produced a number of significant results that contextualize and elaborate upon our primary findings. First, we found that internal motivation—but not the other 2 tested moderators—predicted more initiation of services. For students in the intervention condition, those with high internal motivation were more likely to set up their accounts as well as more likely to initiate other services on campuses by the time of postintervention. For students in the waitlist group (ie, without access to the web-based intervention), those with high internal motivation were even more likely to initiate other campus services, which we might understand as other service initiation in lieu of access to the web-based program. This result further supports our assertion that internal motivation is not sufficient on its own: motivated individuals must have access to an evidence-based intervention in order to improve. Second, the 3 motivation-related constructs as measured at baseline differentially correlated with postintervention variables: intervention adherence was correlated positively with internal motivation and negatively with external motivation; intervention satisfaction was correlated positively with internal motivation and confidence in treatment; and self-report of more intervention barriers correlated negatively with confidence in treatment. These divergent correlational results hint at potential mechanisms, through which internal motivation eventually translates into great symptom reduction. These exploratory results should be replicated by future research that aims to specifically examine associations between internal motivation, service initiation, adherence, and treatment satisfaction.

### Comparison With Prior Work and Future Directions

As reviewed in the introduction, prior studies examining the moderating role of motivation in web-based mental health interventions have produced conflicting results. Some studies found higher motivation predicted more symptom improvement [19], others found higher motivation predicted less symptom improvement [21,22], and some others still found evidence of no significant moderation [19,22]. Across these studies, the various motivation measures included items reflective of internal and external motivation. While the external motivation subscale had low internal consistency, the measure and its supporting theory still conceptually separated 2 conflated constructs of motivation. By separately testing internal and external motivation in this study, the emergence of only 1 moderator as significant—internal motivation—could help explain mixed findings from previous studies as being related to how “motivation” is defined. Another consideration is that our sample consisted of a student population only, which may explain the discrepancy in our results compared to prior research. Future research is encouraged to examine motivation-related constructs in larger and more diverse samples. If the findings of this study are generalizable to other trials of self-guided web-based mental health interventions, then researchers should consider how users with internal motivation might be driving results. Although the intervention produced small effects overall—which is consistent with other self-guided digital interventions [40]—it produced moderate effects for self-motivated individuals. As such, moderator results suggest that overall effects are likely driven by the subset of individuals with high internal motivation. This interpretation is also supported by the finding that students in the waitlist condition with high internal motivation were likely to initiate other services as well as the finding that high internal motivation was more likely among students who have previously or currently used resources. We recommend that researchers conducting such web-based intervention trials measure baseline internal motivation and test it as a moderator of intervention response.

This study improves upon previous research by controlling for baseline symptom severity. First, we would expect those individuals with higher baseline severity to naturally have lower scores at the next assessment period due to the statistical phenomenon of regression toward the mean. Next, symptom severity has been found as a common moderator of effects in other web-based intervention research [41]. Moreover, symptom severity could be a confound or prerequisite of motivation. In terms of the confound risk, there are example items on our selected internal motivation subscale that are conflated with symptom severity (eg, “I feel so guilty about my problems ...”) [17]. We do not believe that there was a fully confounded relationship in this study, as internal motivation predicted greater intervention response over and above baseline symptom severity regardless of condition. However, while severity does not itself guarantee higher internal motivation for an intervention, it may be a prerequisite, in that having some level of symptoms is likely necessary for recognizing a need for change. Thus, it is important to control for baseline severity using measures that separate motivation-related constructs from severity in order to understand their unique contributing roles in intervention response.

Overall, the benefits of intervention uptake and subsequent symptom improvement were primarily experienced by internally motivated individuals. On one hand, these findings suggest that campus services should strategically recruit and target those with internal motivation. On the other hand, a different challenge then remains: How do we help individuals with lower internal motivation at baseline? To develop strategies that enhance baseline internal motivation, future studies could explore predictors of internal motivation to participate in a web-based intervention. First, why are certain populations more likely to have higher internal motivation? Second, how might intervention design features facilitate higher internal motivation? To maximize improvement on web-based interventions, we must first understand and cultivate characteristics such as internal motivation in order to support the success of individuals who might otherwise struggle to improve.

Finally, it would be interesting to investigate why higher internal motivation translates to larger intervention effects. For example, internal motivation could lead to higher adherence (eg, more frequent interaction with the platform), which could in turn lead to better improvement. This hypothesis is feasible, given studies suggesting a dose-response relationship for web-based mental health interventions [42,43]. Moreover, prior research has identified variables similar to internal motivation as significant predictors of adherence to web-based mental health interventions, including intrinsic motivation [22], internal locus of control [44], expected behavioral ability to complete the program [45,46], and self-identification with “preparation” or “action” stages of change [47]. Our exploratory findings already provide preliminary support for the positive relationship between internal motivation and adherence in contrast to external motivation, which was correlated with lower adherence. Such findings suggest not only the importance of differentiating between various types of motivation but also that it would be worthwhile for future research to test adherence as a mediator between internal motivation and intervention effects.

### Limitations

Despite the novelty of differentiating and examining motivation-related constructs as they relate to intervention response, this study has some limitations worthy of consideration. In line with the “law of attrition” and a fundamental challenge of web-based intervention trials [8], a significant limitation in the study is the low postintervention completion rate (ie, 39% attrition). As such, results may be pertinent to the subsample of posttreatment survey completers, limiting the generalizability of our results to the overall sample. Another limitation of this study was that external motivation was not reliably measured using the TMQ subscale. As such, the null moderation results of external motivation in this study cannot be confidently interpreted. Future research is encouraged to explore other more reliable measures of external motivation and examine its relationship with improvement in self-guided web-based interventions. Next, the use of a waitlist condition may have caused some issues. Those assigned to the waitlist condition were notified that they would not be able to access the intervention until the following academic term, which could have been experienced as insensitive or frustrating. If this negative experience worsened their symptom severity or facilitated the increased accessing of other campus services, then it could have contributed to the relative success of the intervention group. An alternative to this waitlist condition would be an active control group such that participants can interact with some kind of parallel platform or materials. A final limitation is the generalizability of our results. This study recruited university students, a population that may have higher internal motivation to engage with new learning of any kind; as such, results may not be translatable to nonstudent populations. Future research should aim to replicate these findings in larger and more diverse populations. Additionally, our web-based intervention was aimed at preventing symptoms of depression and anxiety; thus, results may differ in the context of treatment-level interventions or interventions targeting symptoms that have less overlap with motivational constructs.

### Conclusions

Self-guided web-based programs are often beset with low intervention completion and adherence, whether it be no longer accessing the web-based program midway through delivery or only spending a few minutes passively viewing the intervention content without practicing the learned material [10,48]. Given that the demand for self-motivation has been identified as a main barrier to the web-based intervention tested in this study [16], examining various forms of motivation as potential moderators of intervention effects was warranted. Our findings indicate that a combination of (1) high or moderate internal motivation and (2) access to a self-guided intervention leads to symptom improvement. Considering the implication of internal motivation on intervention outcomes, it is important to understand the characteristics of participants that may best benefit from these interventions and perhaps even create trainings or preintervention courses to cultivate such characteristics. Additional variables related to motivation such as baseline severity, adherence, and motivation-related constructs that reflect individual differences must be explored in order to better understand the moderating role of motivation on a web-based intervention. In conclusion, understanding characteristics that moderate improvement will assist campus services in targeting individuals who can benefit from web-based interventions and can inform treatment strategies that maximize intervention effects.

## Appendix

Multimedia Appendix 1 Adapted Treatment Motivation Questionnaire.

Multimedia Appendix 2 Program Satisfaction Questionnaire.

Multimedia Appendix 3 Digital Intervention Barriers Scale-7.

Multimedia Appendix 4 CONSORT-eHEALTH checklist (V 1.6.1).

## Abbreviations

DASS-21: Depression Anxiety Stress Scale-21
DIBS: Digital Intervention Barriers Scale
LME: linear mixed effects
SDT: self-determination theory
SI: suicidal ideation
TMQ: Treatment Motivation Questionnaire

## Data Availability

The data sets generated and analyzed during this study are available in the Open Science Framework repository [49].
