# Supplementary material for: Motivational Variables as Moderating Effects of a Web-Based Mental Health Program for University Students: Secondary Analysis of a Randomized Controlled Trial
Source: JMIR Form Res. 2024 Jul 3;8:e56118. doi: 10.2196/56118 (PMC11255530; doi:10.2196/56118)
Supplement: Multimedia Appendix 1 [file formative_v8i1e56118_app1.pdf]

Adapted Treatment Motivation Questionnaire

Instructions: Right now you are planning to sign-up for an online program. This questionnaire concerns student's reasons for signing-up and their feelings about the online programming. Different people have different reasons, and we want to know how true each of these reasons is for you.

Please indicate how true each reason is for you, using the following scale:

1	2	3	4	5	6	7
not at all true			somewhat true			very true

<u>I'm signing up for this program because:</u>

1. I really want to make some changes in my life
2. I won't feel good about myself if I don't get some help.
3. I was referred by an advisor, boss, or counselor.
4. I feel so guilty about my problems that I have to do something about it.
5. It is important to me personally to work on my problems.

<u>If I remain in the online program it will probably be because:</u>

6. I'll get in trouble if I don't.
7. I'll feel very bad about myself if I don't.
8. I'll feel like a failure if I don't.
9. I feel like it's the best way to help myself.
10. I don't really feel like I have a choice about staying in the program.
11. I feel it is in my best interests to complete the program.

<u>Rate each of the following in terms of how true each statement is for you.</u>

12. I signed up for this program now because I was under pressure to get help.
13. I am not sure this program will work for me.
14. I am confident this program will work for me.
15. I decided to sign up for this program because I was interested in getting help.
16. I'm not convinced that this program will help me change my habits.
17. I am responsible for choosing to sign-up.
18. I doubt that this program will solve my problems.
19. I chose this program because I think it is an opportunity for change.
20. I am not very confident that I will get results from this program.

<u>Subscales</u>

External Reasons: 3, 6, 10, 12
Internal Reasons: 1, 2, 4, 5, 7, 8, 9, 11, 15, 17, 19
Confidence in Treatment: 13(R), 14, 16(R), 18(R), 20(R)
