# Supplementary material for: Motivational Variables as Moderating Effects of a Web-Based Mental Health Program for University Students: Secondary Analysis of a Randomized Controlled Trial
Source: JMIR Form Res. 2024 Jul 3;8:e56118. doi: 10.2196/56118 (PMC11255530; doi:10.2196/56118)
Supplement: Multimedia Appendix 2 [file formative_v8i1e56118_app2.pdf]

Program Satisfaction Questionnaire

Instructions: Take a moment to consider your responses for each of the following questions. The more informative and honest your responses are, the better able we will be to improve the program in the future.

Q39. How would you rate the quality of the [Redacted] platform and program content?

4 – excellent
3 – good
2 – fair
1 – poor
0 – really bad

Q40. If a friend wanted to learn new skills or form better habits, would you recommend [Redacted] to him or her?

0 – no, definitely not
1 – no, I don't think so
2 – I'm not sure
3 – yes, I think so
4 – yes, definitely

Q41. Did this program help you make progress towards your goal?

4 – yes, definitely
3 – yes, I think so
2 – I'm not sure
1 – no, I don't think so
0 – no, definitely not

Q42. Did this program help you cope more effectively with stress, anxiety, and/or depressed mood?

0 – no, definitely not
1 – no, I don't think so
2 – I'm not sure
3 – yes, I think so
4 – yes, definitely

Q43. Overall, how satisfied are you with [Redacted]?

4 – Very satisfied
3 – Mostly satisfied
2 – Indifferent
1 – Mildly dissatisfied
0 – Very dissatisfied
