# Supplementary material for: Motivational Variables as Moderating Effects of a Web-Based Mental Health Program for University Students: Secondary Analysis of a Randomized Controlled Trial
Source: JMIR Form Res. 2024 Jul 3;8:e56118. doi: 10.2196/56118 (PMC11255530; doi:10.2196/56118)
Supplement: Multimedia Appendix 3 [file formative_v8i1e56118_app3.pdf]

Digital Intervention Barriers Scale (DIBS-7)

Totally disagree 1 2 3 4 5 Totally Agree

1. I had technical problems with the platform
2. I did not understand the platform or things that I was supposed to do on the platform
3. I thought the online intervention was boring
4. I forgot to use the online intervention
5. It was difficult to keep myself motivated to use the intervention
6. The online intervention was available when I needed it
7. I felt that I needed additional support not only from the online intervention, but from a therapist
8. I think the program lasted too long (too many weeks)
9. I think the program was too short (not as many weeks as needed)
10. The online intervention did not seems to be helping me
